# Necrotizing Myopathy Presenting With Guillain-Barré-Like Areflexic Paraparesis, Severe Rhabdomyolysis, and Dialysis-Requiring Acute Kidney Injury

**DOI:** 10.7759/cureus.115560

**Published:** 2026-08-31

**Authors:** Eric J Baghdasaryan, Hasti Soltani, Sari Lada, Chelsea Simpson, Ramesh Nathan

**Affiliations:** 1 Internal Medicine, Los Robles Regional Medical Center, Thousand Oaks, USA; 2 Infectious Disease, Los Robles Regional Medical Center, Thousand Oaks, USA

**Keywords:** acute kidney injury, guillain-barré syndrome mimic, muscle biopsy, necrotizing myopathy, rhabdomyolysis

## Abstract

Necrotizing myopathy typically presents with subacute symmetric proximal weakness and marked creatine kinase elevation. Acute areflexic paraparesis with sensory abnormalities may instead suggest Guillain-Barré syndrome and create uncertainty regarding neuromuscular localization. A man in his 50s presented after collapse with abrupt bilateral lower-extremity paralysis. Examination demonstrated 0/5 strength across proximal and distal lower-extremity muscle groups, marked rigidity and hypercontracture, areflexia, and sensory impairment, with preserved upper-extremity function. Early laboratory abnormalities included serum sodium 164 mmol/L, potassium peaking at 6.5 mmol/L, creatinine 4.96 mg/dL, blood urea nitrogen 194 mg/dL, creatine kinase greater than 60,000 IU/L, and marked aminotransferase elevations. Lumbar puncture on hospital day (HD) 3 showed cerebrospinal fluid protein 70 mg/dL, glucose 66 mg/dL, and fewer than one nucleated cell/mm3. Guillain-Barré syndrome remained an early consideration; intravenous immunoglobulin was administered, and hemodialysis was initiated for severe hyperkalemia in the setting of acute renal failure. Inpatient electromyography and nerve conduction studies were unavailable; therefore, a concurrent neuropathic process could neither be confirmed nor excluded. Bilateral thigh magnetic resonance imaging demonstrated diffuse intramuscular edema, and right-thigh biopsy subsequently established a necrotizing myopathic pattern with frequent necrotic and regenerating myofibers and sparse inflammation. The biopsy pattern was etiologically nonspecific, and the underlying cause remained unresolved. This case illustrates that areflexia and cerebrospinal fluid albuminocytologic dissociation should not override substantial biochemical, radiographic, and histopathologic evidence of muscle injury. In patients with acute paralysis and massive creatine kinase elevation, neuropathic and myopathic processes should be evaluated in parallel.

## Introduction

Myopathies comprise a heterogeneous group of hereditary and acquired skeletal muscle disorders; necrotizing myopathy represents a clinicopathologic pattern characterized by prominent myofiber necrosis and regeneration with relatively sparse inflammation. Importantly, this histopathologic pattern is not synonymous with immune-mediated necrotizing myopathy, as similar necrosis may occur secondary to severe rhabdomyolysis, metabolic or electrolyte disturbances, and other systemic insults. Immune-mediated necrotizing myopathy may be associated with antibodies against signal recognition particle (SRP) or 3-hydroxy-3-methylglutaryl-coenzyme A reductase (HMGCR), whereas similar necrotizing patterns may also occur with medications, toxins, infections, malignancy, endocrine disease, and severe systemic metabolic stress [1,2]. Patients most commonly present with symmetric proximal weakness and marked elevation of serum creatine kinase, although the clinical phenotype and severity vary considerably [1,2].

Acute weakness accompanied by areflexia and cerebrospinal fluid albuminocytologic dissociation often raises concern for Guillain-Barré syndrome. The diagnosis is primarily clinical and is supported by cerebrospinal fluid analysis and electrodiagnostic testing [3,4]. However, massive creatine kinase elevation, severe rhabdomyolysis, and associated acute kidney injury are not adequately explained by an isolated demyelinating polyradiculoneuropathy and should prompt evaluation for a concurrent or alternative muscle disorder [5-7].

We report a man in his 50s who presented with abrupt bilateral lower-extremity paralysis, areflexia, sensory abnormalities, profound volume depletion, severe rhabdomyolysis, and dialysis-requiring acute kidney injury. Although the initial clinical and cerebrospinal fluid findings suggested Guillain-Barré syndrome, subsequent muscle imaging and biopsy demonstrated a necrotizing myopathic process. The case illustrates the importance of separating neuromuscular localization from etiologic diagnosis when examination findings and objective markers of tissue injury are discordant.

## Case presentation

A man in his 50s presented after collapsing in a public setting. He reported preceding lightheadedness but denied loss of consciousness, head trauma, chest pain, dyspnea, gastrointestinal symptoms, or recent infectious illness. At the time of collapse, he experienced abrupt loss of motor control in both lower extremities.

Initial neurologic examination demonstrated complete bilateral lower-extremity immobility, with 0/5 strength across both proximal and distal muscle groups. Lower-extremity tone was markedly increased, with prominent rigidity and hypercontracture rather than flaccidity. Deep tendon reflexes were absent in the lower extremities, and sensation was decreased bilaterally without a sensory level. Upper-extremity strength, tone, sensation, and reflexes were preserved. Cranial nerve examination and bulbar function were intact. Because of the severity of the paraparesis, the patient was unable to stand or ambulate, precluding formal gait assessment.

On presentation, the patient was tachycardic at 139 beats/min and hypotensive at 90/54 mm Hg. Laboratory testing was obtained during the initial evaluation in the setting of collapse, profound weakness, and acute renal failure rather than specifically as part of the subsequent evaluation for Guillain-Barré syndrome. Initial and early abnormalities included serum sodium 164 mmol/L, hemoglobin 20.5 g/dL, lactate 7.9 mmol/L, blood urea nitrogen 194 mg/dL, creatinine 4.96 mg/dL, bicarbonate 14 mmol/L, anion gap 21, and creatine kinase greater than 60,000 IU/L. Admission aspartate aminotransferase was 33 IU/L and alanine aminotransferase was 59 IU/L; both increased markedly during the early hospitalization. Urinalysis was positive for blood; urine myoglobin was not measured. Crystalloid volume resuscitation was initiated with 1 L of normal saline and 1 L of lactated Ringer solution, with improvement in blood pressure.

Hypernatremia was present on admission, with serum sodium 164 mmol/L. Serial sodium measurements were 163 mmol/L later on hospital day (HD) 1, 153 and 141 mmol/L on HD 2, 139 mmol/L on HD 3, 141 and 143 on HD 4.

Baseline renal function was not available. Potassium subsequently increased to 6.5 mmol/L despite initial medical management, prompting initiation of intermittent hemodialysis on HD 2 for severe hyperkalemia in the setting of acute renal failure. No specific electrocardiographic manifestations attributable to hyperkalemia were documented. Renal dysfunction persisted, with creatinine reaching 9.05 mg/dL during the hospitalization, and the patient became anuric during the dialysis-dependent phase. Urine output subsequently recovered. Laboratory trajectories by hospital day are shown in Table 1.

**Table 1:** Selected laboratory trajectories by hospital day. Abbreviations: ALT, alanine aminotransferase; AST, aspartate aminotransferase; BUN, blood urea nitrogen; HD, hospital day. Dashes indicate values not selected for this summary table.

Laboratory test	HD 1 (admission/early)	HD 3-4	HD 8-10	HD 12-13	HD 25 (discharge)
Sodium, mmol/L	164, then 163	139 (HD 3); 141 and 143 (HD 4)	-	-	143
Potassium, mmol/L	3.8; peak 6.5 on HD 2	-	-	-	3.3
BUN, mg/dL	194	113 (HD 3); 93 (HD 4)	49 (HD 8); 40 (HD 9); 58 (HD 10)	117 (HD 12); 77 (HD 13)	64
Creatinine, mg/dL	4.96	4.89 (HD 3); 5.53 (HD 4)	5.38 (HD 8); 4.24 (HD 9); 5.60 (HD 10)	9.05 (HD 12); 5.97 (HD 13)	2.75
Bicarbonate, mmol/L	14	-	-	-	29
Lactate, mmol/L	7.9	-	-	-	-
Creatine kinase, IU/L	>60,000 (early)	-	11,578 (HD 8); 3,807 (HD 9); 1,683 (HD 10)	-	-
AST, IU/L	33	5,102 (HD 3); 5,134 (HD 4)	1,466 (HD 8); 845 (HD 9)	143 (HD 12); 111 (HD 13)	33
ALT, IU/L	59	967 (HD 3); 1,208 (HD 4)	900 (HD 8); 811 (HD 9)	312 (HD 12); 258 (HD 13)	47
Hemoglobin, g/dL	20.5	-	-	-	-
Urine output	Anuria on arrival	-	Anuria during dialysis-dependent phase	-	>2 L/24 h

Central and spinal structural causes of the acute paraparesis were investigated with neuroimaging. Brain MRI demonstrated no acute intracranial abnormality, including no acute infarction, hemorrhage, or mass lesion. MRI of the cervical spine showed only mild degenerative disc disease without spinal canal stenosis, neural foraminal narrowing, or abnormal spinal cord signal. Thoracic spine MRI was unremarkable. Lumbar spine MRI demonstrated only minimal degenerative disc disease without spinal canal stenosis or neuroforaminal narrowing. These studies identified no central or spinal structural lesion sufficient to account for the acute bilateral lower-extremity paralysis.

Lumbar puncture on HD 3 demonstrated cerebrospinal fluid protein of 70 mg/dL, glucose of 66 mg/dL, fewer than one nucleated cell/mm3, and six red blood cells/mm3, consistent with albuminocytologic dissociation. Given the acute bilateral weakness, areflexia, sensory impairment, and cerebrospinal fluid findings, Guillain-Barré syndrome remained an early diagnostic consideration. Inpatient electromyography and nerve conduction studies were not available at our institution and therefore were not performed during the hospitalization; outpatient electrodiagnostic evaluation was recommended.

Because Guillain-Barré syndrome remained a clinically important possibility, intravenous immunoglobulin was administered at 25 g daily for five doses (cumulative dose, 125 g), corresponding to approximately 0.4 g/kg/day and an approximately 2 g/kg cumulative course based on contemporaneous body weight. Plasma exchange was also considered as an alternative if long-term dialysis dependence persisted; ultimately, intravenous immunoglobulin was administered as the initial immunotherapy for the suspected Guillain-Barré syndrome. Serial neurologic examinations demonstrated partial improvement following intravenous immunoglobulin therapy, with lower-extremity strength improving from 0/5 to approximately 2/5, return of reduced reflexes, and improvement in sensation. Upper-extremity function remained preserved.

The magnitude of creatine kinase elevation, rhabdomyolysis, and persistent weakness prompted evaluation for a primary or secondary muscle disorder. Creatine kinase declined to 11,578 IU/L on HD 8, 3,807 IU/L on HD 9, and 1,683 IU/L on HD 10. Aldolase was elevated at 49.5 U/L. Bilateral thigh MRI on HD 13 demonstrated extensive bilateral subcutaneous edema and diffuse intramuscular edema, predominantly involving the quadriceps with lesser hamstring involvement. Interstitial fluid tracked along several myotendinous junctions and was most pronounced near the gracilis muscles. No discrete abscess, fracture, or infiltrative lesion was identified. Representative coronal, long-axis, and axial images demonstrating the bilateral distribution, craniocaudal extent, and multicompartment involvement are shown in Figures 1-4. The findings demonstrated extensive muscle injury but were not etiologically specific.

**Figure 1:**
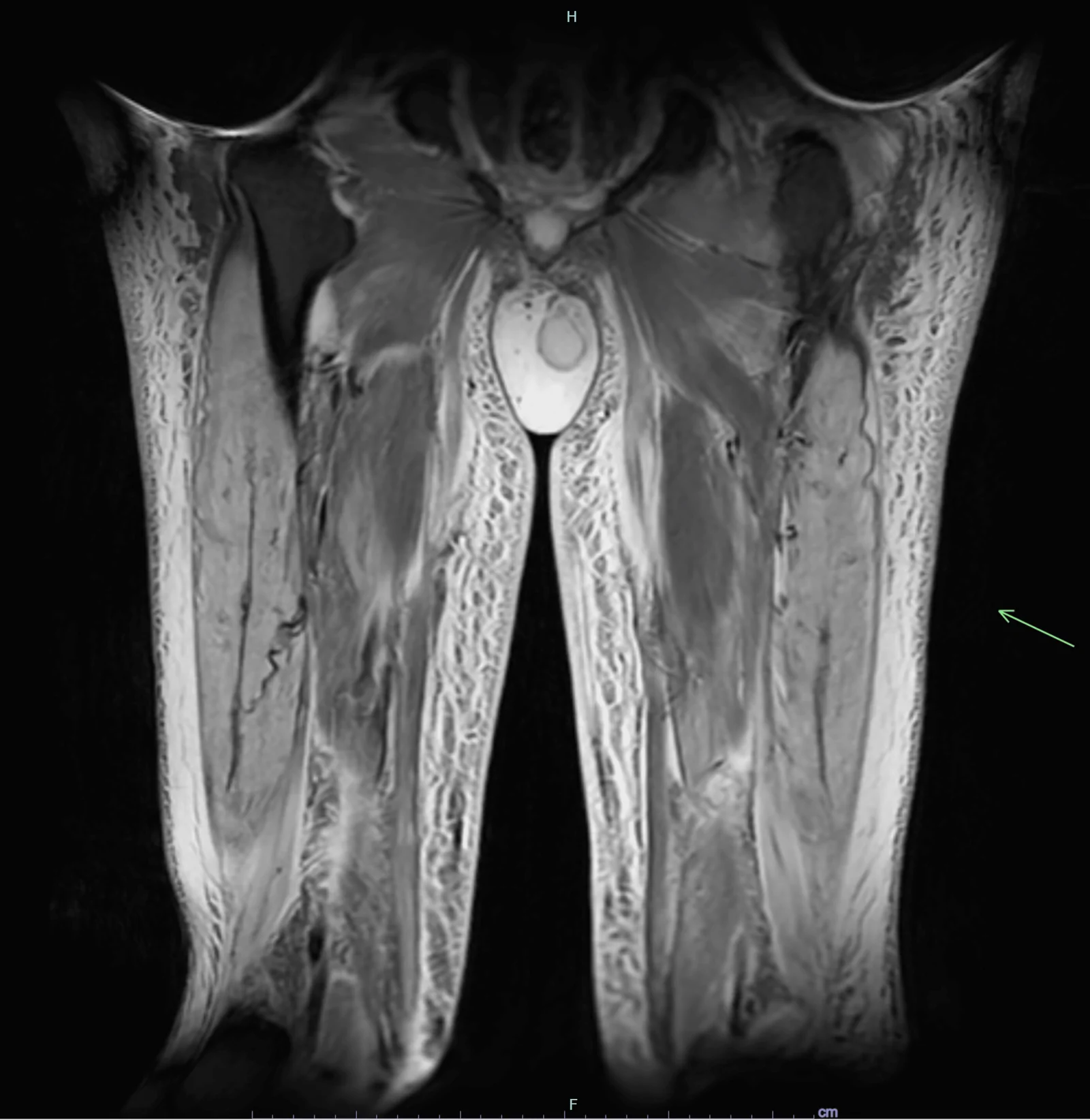
Coronal MRI Demonstrating Bilateral Thigh Muscle Edema Noncontrast coronal MR image of both thighs demonstrates extensive bilateral subcutaneous edema and patchy intramuscular signal abnormality involving multiple muscle groups, illustrating the bilateral distribution of the process.

**Figure 2:**
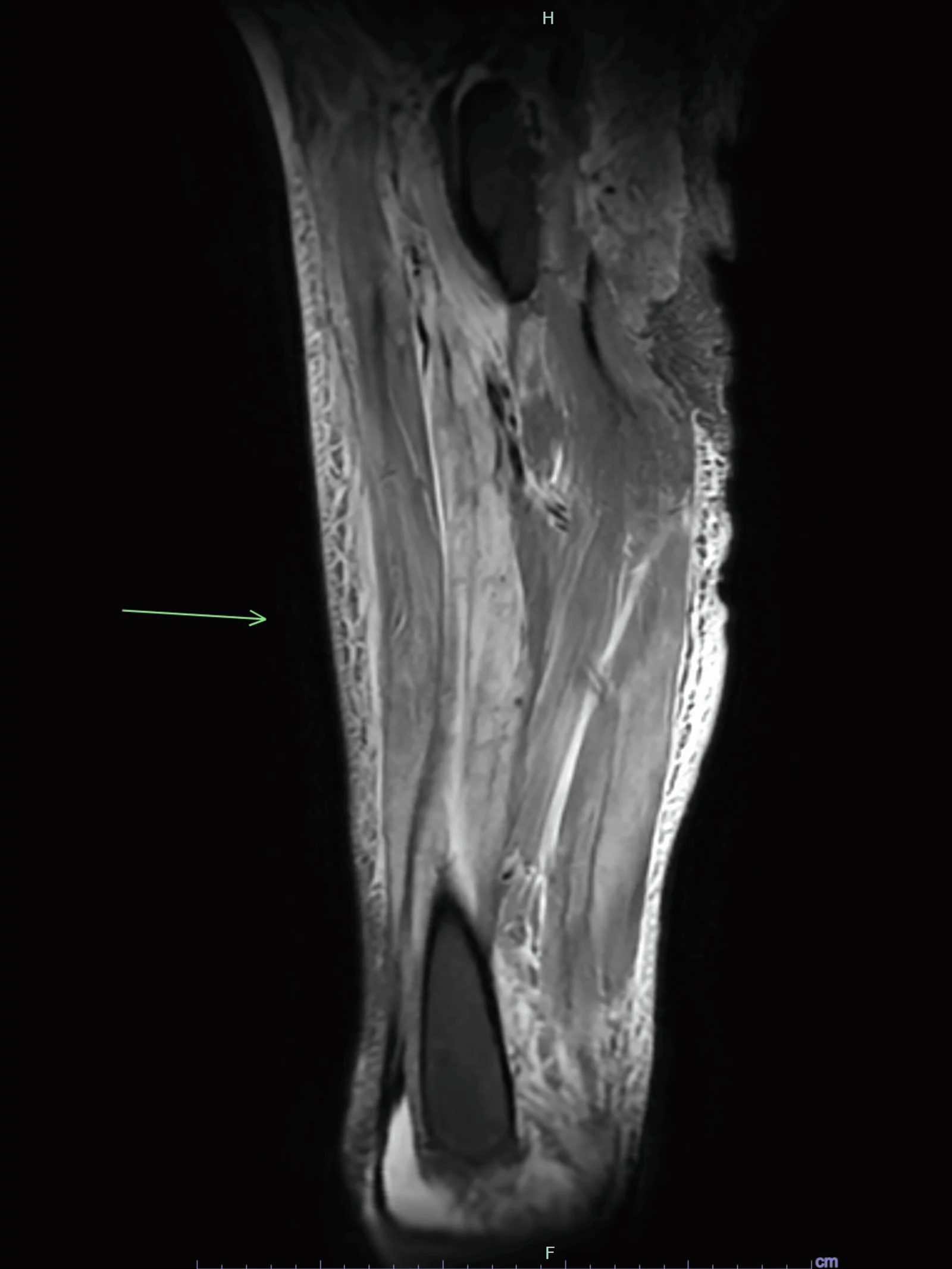
Long-Axis Thigh MRI Demonstrating the Craniocaudal Extent of Edema Noncontrast long-axis MR image demonstrates extensive longitudinal intramuscular and subcutaneous edema through a substantial portion of the thigh, illustrating the diffuse rather than focal nature of the process.

**Figure 3:**
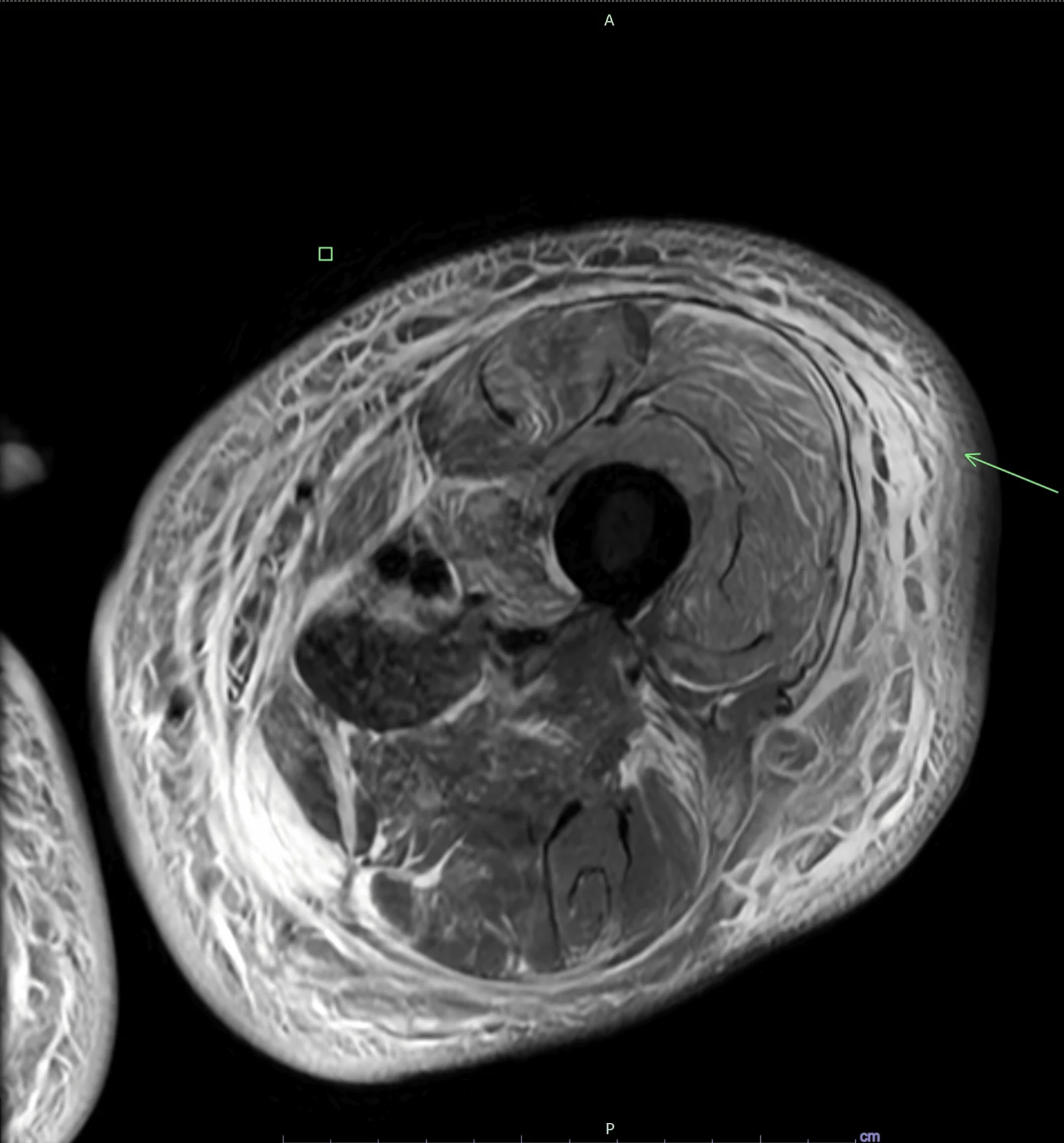
Axial MRI demonstrating multicompartment thigh involvement. Noncontrast axial MR image through the thighs demonstrates diffuse subcutaneous edema and heterogeneous intramuscular signal abnormality involving multiple muscle compartments.

**Figure 4:**
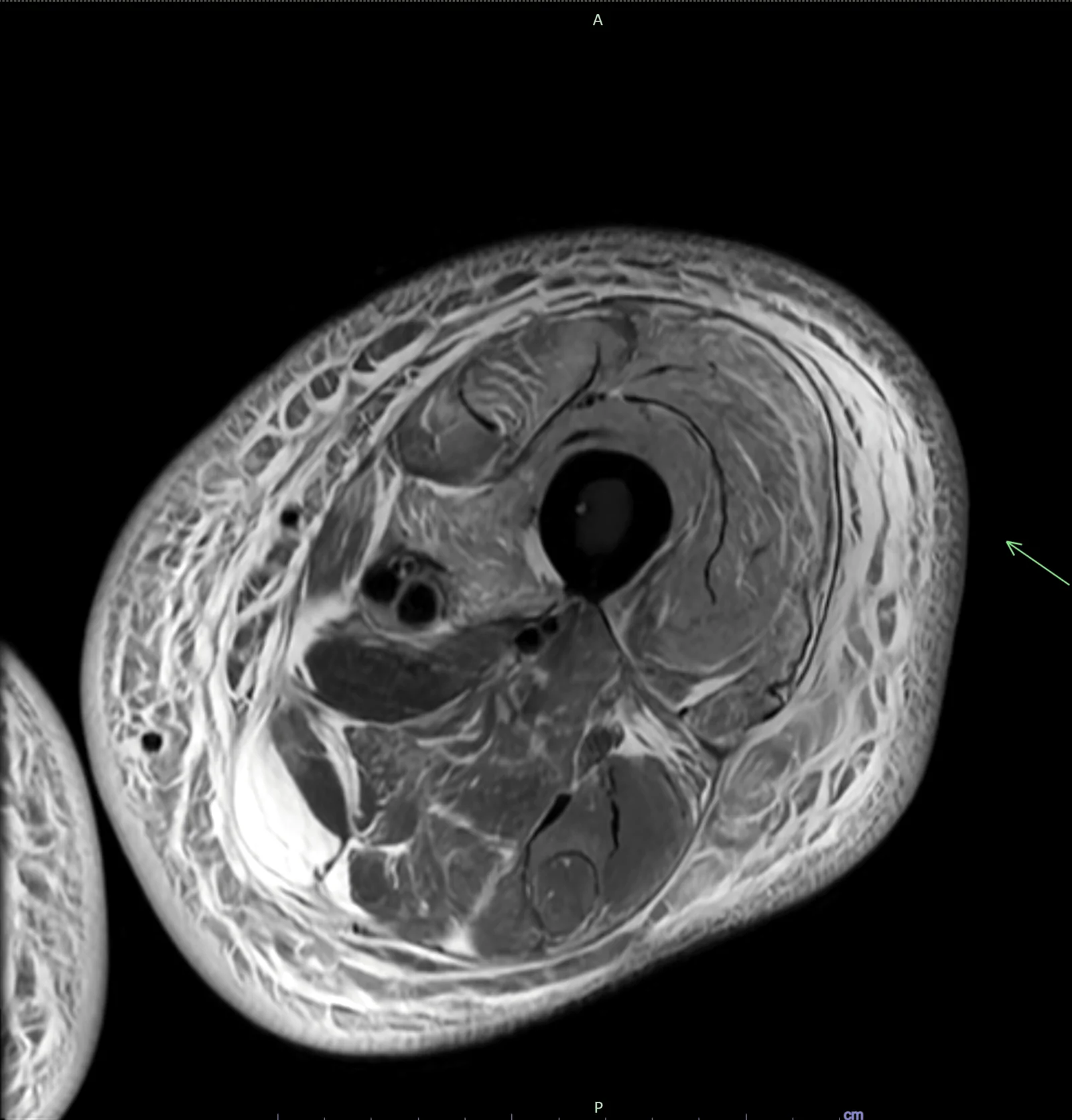
Axial MRI at a different thigh level demonstrating widespread muscle edema. Noncontrast axial MR image obtained at a different thigh level demonstrates circumferential subcutaneous edema with heterogeneous signal abnormality involving anterior, medial, and posterior muscle compartments, confirming widespread multicompartment involvement.

After thigh MRI demonstrated extensive intramuscular edema concerning for myositis, intravenous methylprednisolone 1 g daily was initiated on HD 13 for five days. Corticosteroid therapy was therefore directed at the suspected myopathic process rather than the previously considered Guillain-Barré syndrome. Further gradual clinical improvement was observed during corticosteroid therapy, although substantial bilateral lower-extremity weakness persisted. The patient was subsequently transitioned to prednisone 60 mg daily, with a planned taper in 10-mg increments at approximately two-week intervals.

The etiologic evaluation did not establish a specific cause of the necrotizing myopathic pattern. Anti-HMGCR antibodies, anti-SRP antibodies, and an extended myositis-specific/myositis-associated antibody panel were not performed; therefore, immune-mediated necrotizing myopathy could not be serologically confirmed or excluded. Available autoimmune testing, including double-stranded DNA antibody, antinuclear antibody, and antineutrophil cytoplasmic antibodies, was negative. Thyroid and cortisol testing were normal. Toxicologic evaluation and alcohol testing were negative. No home medications were documented on admission, and no preadmission statin, nonprescription medication, supplement, or herbal-product exposure was identified. The patient denied a recent infectious illness. HIV and viral hepatitis testing were negative, and blood cultures were negative. Malignancy evaluation, including hematology/oncology assessment and serum CA 19-9, CEA, prostate-specific antigen (PSA), and alpha-fetoprotein (AFP) testing, did not identify an underlying malignancy; serum protein electrophoresis was unremarkable. No vitamin B12 deficiency was identified; folate was reduced at 2.9 ng/mL and was treated with supplementation. No preceding prolonged immobilization, seizure activity, heat exposure, or strenuous exertional activity was identified.

An open biopsy of the right thigh was performed on HD 19 after MRI had demonstrated extensive bilateral thigh muscle involvement. The biopsy was obtained after the five-day course of high-dose intravenous methylprednisolone. Histopathologic examination demonstrated a patchy to nearly diffuse distribution of frequent necrotic and regenerating myofibers with a subacute monophasic appearance and only very limited chronic lymphoid inflammation, establishing a necrotizing myopathic pattern. The final pathology interpretation emphasized that this pattern was etiologically nonspecific. Immune-mediated necrotizing myopathy and a partially treated inflammatory myopathy were considered but were regarded as less likely on morphologic grounds, particularly given the geographic pattern of myofiber injury and the severity of creatine kinase elevation. Enzyme histochemical evaluation showed no diagnostic features of a specific glycogen, lipid, or mitochondrial myopathy, and no morphologic evidence of vasculitis was identified. Histopathologic findings are demonstrated in Figure 5.

**Figure 5:**
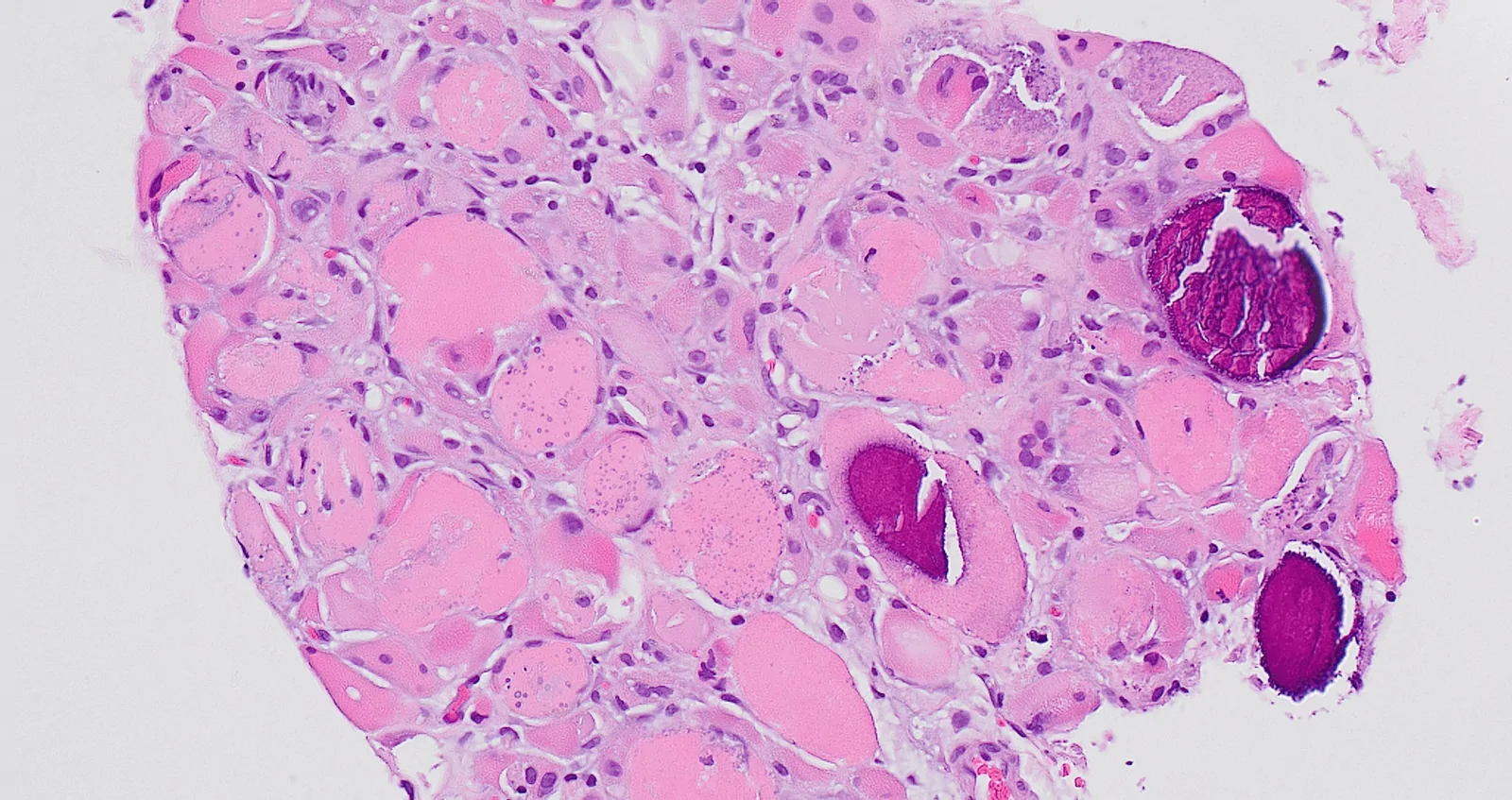
Necrotizing Myopathy With Regeneration and Dystrophic Calcification Hematoxylin and eosin-stained right-thigh skeletal muscle demonstrates marked variation in fiber size with numerous necrotic and regenerating myofibers, scattered angular and severely atrophic fibers, increased internal nuclei, and focal dystrophic calcification. Chronic inflammation is sparse, and no vasculitis is identified. Although angular and severely atrophic fibers may raise consideration of a neurogenic component, the final pathology interpretation did not establish denervation or neurogenic atrophy; these findings were therefore considered nonspecific in this case.

Near discharge, renal function had improved but remained abnormal. Outpatient hemodialysis was arranged with nephrology follow-up pending further assessment of renal recovery. Dedicated neurologic examinations late in the hospitalization continued to show severe lower-extremity weakness, approximately 2/5 proximally greater than distally, with reduced reflexes and improved sensation, while upper-extremity function remained preserved. Because of persistent functional impairment, the patient was discharged to a supported postacute setting for continued physical and occupational rehabilitation, with outpatient nephrology, neurology, and rheumatology follow-up and the planned prednisone taper.

The principal clinical events, neurologic findings, laboratory trajectories, diagnostic studies, and therapeutic interventions are summarized by hospital day in Table 2.

**Table 2:** Clinical course by hospital day. HD, hospital day; HD 1 denotes the day of admission. ALT, alanine aminotransferase; AST, aspartate aminotransferase; BUN, blood urea nitrogen; CK, creatine kinase; AIDP, acute inflammatory demyelinating polyradiculoneuropathy; IVIG, intravenous immunoglobulin; LP, lumbar puncture; AKI, acute kidney injury.

Hospital day	Clinical findings / investigations	Laboratory / renal course	Interventions
HD 1	Collapse preceded by lightheadedness with abrupt loss of bilateral lower-extremity motor control. Marked rigidity/hypercontracture; profound motor impairment.	Na 164 mmol/L; K 3.8 mmol/L initially; BUN 194 mg/dL; creatinine 4.96 mg/dL; lactate 7.9 mmol/L; bicarbonate 14 mmol/L; AST 33 IU/L; ALT 59 IU/L; CK >60,000 IU/L (early); hemoglobin 20.5 g/dL.	Immediate crystalloid volume resuscitation with improvement in blood pressure.
HD 2	Persistent profound lower-extremity motor impairment.	Potassium peaked at 6.5 mmol/L with severe AKI and metabolic abnormalities.	Temporary dialysis access placed; intermittent hemodialysis initiated for severe hyperkalemia in acute renal failure.
HD 3	Formal neurologic examination: 0/5 bilateral lower-extremity strength, areflexia, decreased sensation; upper extremities preserved. Brain MRI without acute infarction, hemorrhage, or mass lesion. LP showed protein 70 mg/dL, glucose 66 mg/dL, <1 nucleated cell/mm3, 6 RBC/mm3.	Na 139 mmol/L; BUN 113 mg/dL; creatinine 4.89 mg/dL; AST 5,102 IU/L; ALT 967 IU/L.	Neurology considered AIDP/Guillain-Barré syndrome.
HD 5-9	Serial neurologic examinations showed partial improvement to approximately 2/5 lower-extremity strength, return of reduced reflexes, and improved sensation; upper extremities preserved.	CK declined to 11,578 IU/L (HD 8) and 3,807 IU/L (HD 9). Renal dysfunction persisted with ongoing hemodialysis.	IVIG 25 g daily for five doses (cumulative 125 g) for suspected Guillain-Barré syndrome. Plasma exchange was considered as an alternative if long-term dialysis dependence persisted.
HD 10-11	Lower-extremity strength remained approximately 2/5, proximal greater than distal, with reduced reflexes and improved sensation. Cervical, thoracic, and lumbar MRI showed no explanatory spinal cord or compressive lesion.	CK 1,683 IU/L (HD 10). Creatinine 5.60 mg/dL (HD 10) and 6.89 mg/dL (HD 11).	Continued renal replacement and supportive care.
HD 13	Severe lower-extremity weakness persisted. Bilateral thigh MRI demonstrated extensive subcutaneous and diffuse intramuscular edema, predominantly quadriceps, with lesser hamstring involvement and myotendinous fluid tracking.	Creatinine 5.97 mg/dL; AST 111 IU/L; ALT 258 IU/L.	IV methylprednisolone 1 g daily initiated for suspected inflammatory/myopathic process.
HD 13-17	Gradual neurologic improvement continued, but substantial weakness persisted.	CK and aminotransferases continued to decline; renal dysfunction persisted.	Methylprednisolone 1 g daily for five days.
HD 18	Persistent severe lower-extremity weakness.	-	Transitioned to prednisone 60 mg daily.
HD 19	Persistent motor impairment.	-	Open right-thigh muscle biopsy.
HD 20-25	Dedicated neurologic examinations continued to show severe lower-extremity weakness with preserved upper-extremity function; dependent mobility requiring rehabilitation.	Renal function improved but remained abnormal. At discharge: BUN 64 mg/dL, creatinine 2.75 mg/dL, bicarbonate 29 mmol/L, urine output >2 L/24 h.	Discharged to supported postacute setting with outpatient hemodialysis, rehabilitation, specialty follow-up, and prednisone taper.

## Discussion

The distinction between localization and etiology is central to this case. The marked creatine kinase elevation, severe rhabdomyolysis, diffuse thigh muscle edema, and biopsy-confirmed myofiber necrosis establish substantial skeletal muscle injury; however, they do not establish the cause of that injury or prove that the entire neurologic syndrome originated exclusively in muscle. The necrotizing myopathic pattern could reflect a primary immune-mediated process, secondary myofiber necrosis associated with severe rhabdomyolysis, toxic-metabolic or electrolyte-associated injury, ischemic injury, or overlapping mechanisms. At the same time, the accompanying sensory impairment and areflexia raise the possibility of a concurrent peripheral neuropathic process. Because inpatient electrodiagnostic testing was unavailable, a combined neuropathic and myopathic disorder could neither be confirmed nor excluded.

Guillain-Barré syndrome was a reasonable initial diagnostic consideration because of the acute bilateral lower-extremity weakness, areflexia, sensory impairment, and cerebrospinal fluid findings. Lumbar puncture on HD 3 demonstrated protein 70 mg/dL with fewer than one nucleated cell/mm3, consistent with albuminocytologic dissociation. However, this finding is not specific for Guillain-Barré syndrome [3,4]. Inpatient electromyography and nerve conduction studies were unavailable and were therefore deferred to outpatient follow-up. Consequently, the available data could not distinguish among an acute inflammatory demyelinating or axonal neuropathic process, a primary myopathic process, or combined neuropathic and myopathic disease. Guillain-Barré syndrome therefore remained a clinical consideration rather than a confirmed diagnosis.

The magnitude of creatine kinase elevation was particularly difficult to reconcile with an isolated demyelinating polyradiculoneuropathy. Creatine kinase elevation has been reported in Guillain-Barré syndrome, particularly with acute motor axonal neuropathy, but reported elevations are generally substantially lower than that observed in this patient [7]. The accompanying rhabdomyolysis, marked aminotransferase elevations, diffuse thigh muscle edema, and biopsy-proven myofiber necrosis established a major myopathic process but did not exclude a concurrent neuropathy. Massive creatine kinase elevation should therefore be interpreted as a localization signal rather than as an incidental complication of acute weakness.

Magnetic resonance imaging provided an important bridge between the biochemical findings and tissue diagnosis. Extensive bilateral intramuscular edema confirmed widespread muscle involvement, but muscle edema is sensitive rather than etiologically specific and may occur with immune-mediated myopathy, rhabdomyolysis, infection, trauma, denervation, or metabolic injury [8]. Imaging therefore established the distribution and severity of muscle involvement but not its cause.

The final pathologic interpretation further favored caution in assigning an immune-mediated etiology. Histology demonstrated frequent necrotic and regenerating myofibers with sparse chronic inflammation, establishing a necrotizing myopathic pattern. Although immune-mediated necrotizing myopathy and partially treated inflammatory myopathy were considered, they were regarded as less likely on morphologic grounds because of the geographic pattern of myofiber injury and the severity of creatine kinase elevation. No morphologic or enzyme-histochemical features diagnostic of a specific glycogen, lipid, or mitochondrial myopathy were identified, and there was no morphologic evidence of vasculitis. The biopsy therefore established localization to skeletal muscle and a necrotizing pattern but did not establish a specific cause. Because the biopsy followed both intravenous immunoglobulin and high-dose corticosteroid therapy, attenuation of inflammatory features by preceding treatment cannot be excluded; sparse inflammation should therefore not be interpreted as definitive evidence against a previously more inflammatory process. Scattered angular and severely atrophic fibers raised the possibility of a neurogenic contribution; however, the pathology report did not identify these findings as sufficient to establish denervation. In the absence of inpatient electrodiagnostic testing, a concurrent neurogenic process remained unresolved.

Immune-mediated necrotizing myopathy remained within the differential diagnosis but was not established. Anti-HMGCR antibodies, anti-SRP antibodies, and an extended myositis-specific/myositis-associated antibody panel were not performed; accordingly, the available evaluation cannot classify the process as antibody-positive or seronegative immune-mediated necrotizing myopathy. No preadmission statin or other medication exposure was identified, and the available autoimmune, toxicologic, infectious, endocrine, and malignancy evaluations did not reveal a specific alternative etiology. Paraneoplastic disease was considered within the differential, but inpatient hematology/oncology assessment and available imaging and serum testing did not identify an underlying malignancy. The documented folate deficiency was treated but was not considered sufficient to explain the magnitude of muscle injury. No preceding seizure, prolonged immobilization, heat exposure, or exertional injury was identified. In the setting of profound volume depletion, renal failure, marked electrolyte and metabolic abnormalities, and severe rhabdomyolysis, a toxic-metabolic or secondary necrotizing process remained plausible; however, a specific causal mechanism could not be established.

The acute kidney injury was likely multifactorial. Profound volume depletion plausibly reduced renal perfusion, whereas severe rhabdomyolysis may have contributed through pigment-mediated tubular injury [5,6]. Urine myoglobin was not measured, and renal biopsy was not performed; therefore, pigment nephropathy was not directly confirmed. The patient developed severe hyperkalemia and dialysis-dependent acute renal failure, including a period of anuria, before urine output and renal function partially recovered. At discharge, urine output exceeded 2 L/24 hours and creatinine had improved to 2.75 mg/dL, but renal recovery remained incomplete and outpatient hemodialysis had been arranged pending continued nephrology assessment. Long-term renal outcome was unavailable.

The temporal course does not permit attribution of recovery to a single intervention. Partial neurologic improvement was documented before corticosteroid initiation, and additional gradual clinical improvement was observed during corticosteroid therapy; however, severe lower-extremity weakness persisted. Similarly, the decline in creatine kinase occurred in the setting of concurrent volume resuscitation, metabolic correction, renal replacement therapy, intravenous immunoglobulin, corticosteroid treatment, and supportive care. Accordingly, neither the biochemical improvement nor the neurologic trajectory can be considered therapeutic confirmation of an immune-mediated etiology. Plasma exchange was considered but was not pursued. For Guillain-Barré syndrome, intravenous immunoglobulin and plasma exchange are both established first-line immunotherapies, without evidence supporting routine preference for plasma exchange over intravenous immunoglobulin [4]. The mixed neuropathic-myopathic phenotype itself did not establish an indication for plasma exchange, particularly because the etiology of the muscle injury remained uncertain. Selection of intravenous immunoglobulin should therefore be interpreted as empiric treatment of the suspected neuropathic component rather than treatment of a confirmed unified immune-mediated neuromuscular disorder.

This case has several important limitations. Electrodiagnostic studies were unavailable during the hospitalization, preventing definitive characterization of a demyelinating, axonal, myopathic, or mixed neuromuscular process. Anti-HMGCR, anti-SRP, and an extended myositis antibody panel were not performed. Muscle biopsy was obtained after immunotherapy, which may have altered inflammatory features. Urine myoglobin was not measured and renal biopsy was not performed. Long-term neurologic and renal outcomes were unavailable. These limitations prevent definitive attribution of the weakness to a single disorder and preclude determination of the underlying cause of the necrotizing myopathic pattern.

The principal lesson is not that Guillain-Barré syndrome and necrotizing myopathy necessarily coexisted. Rather, the case demonstrates that a Guillain-Barré-like presentation should not establish neuropathic localization when substantial biochemical, radiographic, and histopathologic evidence of muscle injury is present. Extreme creatine kinase elevation, severe rhabdomyolysis, extensive muscle edema, or rhabdomyolysis-associated acute kidney injury should prompt parallel evaluation for primary and secondary muscle disease. Early integration of the neurologic examination with biochemical trends, muscle imaging, electrodiagnostic testing, serologic studies, and biopsy may reduce diagnostic anchoring and clarify whether neuropathic and myopathic processes coexist.

## Conclusions

Acute areflexic weakness with elevated cerebrospinal fluid protein may suggest Guillain-Barré syndrome, but these findings are not specific. In this patient, the biochemical, radiographic, and histopathologic findings established a major necrotizing muscle process, but neither its etiology nor its relationship to the entire neurologic syndrome could be definitively determined. Guillain-Barré syndrome was not confirmed because inpatient electrodiagnostic testing was unavailable, and a concurrent neuropathic process could neither be confirmed nor excluded. When acute paralysis is accompanied by substantial biochemical or radiographic evidence of muscle injury, clinicians should evaluate neuropathic and myopathic diagnoses in parallel rather than force discordant findings into a single initial diagnosis.
